# Potential for Zoonotic Transmission of *Brachyspira pilosicoli*

**DOI:** 10.3201/eid1205.051180

**Published:** 2006-05

**Authors:** David J. Hampson, Sophy L. Oxberry, Tom La

**Affiliations:** * Murdoch University, Murdoch, Western Australia, Australia

**Keywords:** zoonoses, Brachyspira pilosicoli, letter

**To the Editor:** Anaerobic intestinal spirochetes of the genus *Brachyspira* colonize the large intestine (*1*). Most *Brachyspira* species have a restricted host range, whereas *Brachyspira* (formerly *Serpulina*) *pilosicoli* colonizes a variety of animal and bird species and humans. *B. pilosicoli* is an important colonic pathogen of pigs and chickens (*2*). It occurs at high prevalence rates in humans in developing countries and in male homosexuals and HIV-positive persons in industrialized countries (*3*). Its potential as a human pathogen was emphasized after its identification in the bloodstream of a series of debilitated persons (*4*).

*B. pilosicoli* isolates from humans and other species have been used experimentally to colonize chicks, piglets, and mice (*5**–**7*). While these results indicate that the *B. pilosicoli* strains used lacked host-species specificity, few data exist on whether natural zoonotic spread of *B. pilosicoli* strains occurs. In 1 study that used pulsed-field gel electrophoresis (PFGE) to type isolates from Papua New Guinea, 2 dogs were colonized with *B. pilosicoli* isolates with the same PFGE types as those from villagers. However, the higher prevalence of colonization with *B. pilosicoli* in humans than dogs suggested that the dogs were infected with human isolates, probably through consumption of human feces (*8*).

Multilocus enzyme electrophoresis (MLEE) has been used to study variation in *B. pilosicoli* isolates; most studies have focused on isolates from only 1 or 2 host species (*8**–**10*). Generally, *B. pilosicoli* isolates are diverse, and a lack of linkage disequilibrium in the MLEE data for human isolates suggests that the species is recombinant (*8*).

We used MLEE to investigate relationships between 107 *B. pilosicoli* isolates of diverse geographic and host-species origins and the *B. aalborgi* type strain (NCTC 11492^T^). Isolates were selected on the basis of their diverse origins and availability in the Murdoch University culture collection. They originated from feces of 34 pigs, 19 chickens, 13 ducks, 1 rhea, 25 humans, and 4 dogs; from 7 human blood samples; and from 4 water sources frequented by waterfowl. Isolates originated from Australia, Canada, France, Italy, the Netherlands, Oman, Papua New Guinea, the United Kingdom, and the United States.

The MLEE method used was as previously described (*8**–**10*); the electrophoretic mobility of 15 constitutive enzymes was analyzed. Variations in electrophoretic mobility were interpreted as representing products of different alleles at each enzyme locus. Isolates with identical enzymatic profiles at 15 loci were grouped into an electrophoretic type (ET). Genetic distance between ETs was calculated as the proportions of loci at which dissimilar alleles occurred. PHYLIP version 3.51c (Phylogeny Inference Package, University of Washington, Seattle, WA, USA) was used to analyze data and generate a dendrogram by using the unweighted pair-group method with arithmetic mean clustering fusion strategy. Genetic diversity (h) was calculated for the number of ETs as (1 – Σpi2)(n/n – 1), where pi is the frequency of the indicated allele and n is the number of ETs.

*B. pilosicoli* isolates were divided into 80 ETs (mean 1.35 isolates per ET) (Figure). *B. aalborgi* NTCC 11492^T^ was distinct in ET81. The *B. pilosicoli* isolates were diverse, with an h value of 0.41. Generally, they did not cluster according to host species of origin, and isolates from a given species were distributed throughout the dendrogram. Isolates from birds were more diverse than those from humans and pigs. Eight ETs contained multiple isolates, in each case from the same host species (either chickens or pigs). In 4 cases these originated from different countries: ET47 contained 2 Australian porcine isolates and 2 from the United States; ET53 contained 2 Australian porcine isolates and Scottish porcine type strain P43/6/78^T^; ET54 contained 2 Australian and 2 Canadian porcine isolates; ET65 contained 1 Dutch and 1 US chicken isolate.

**Figure:**
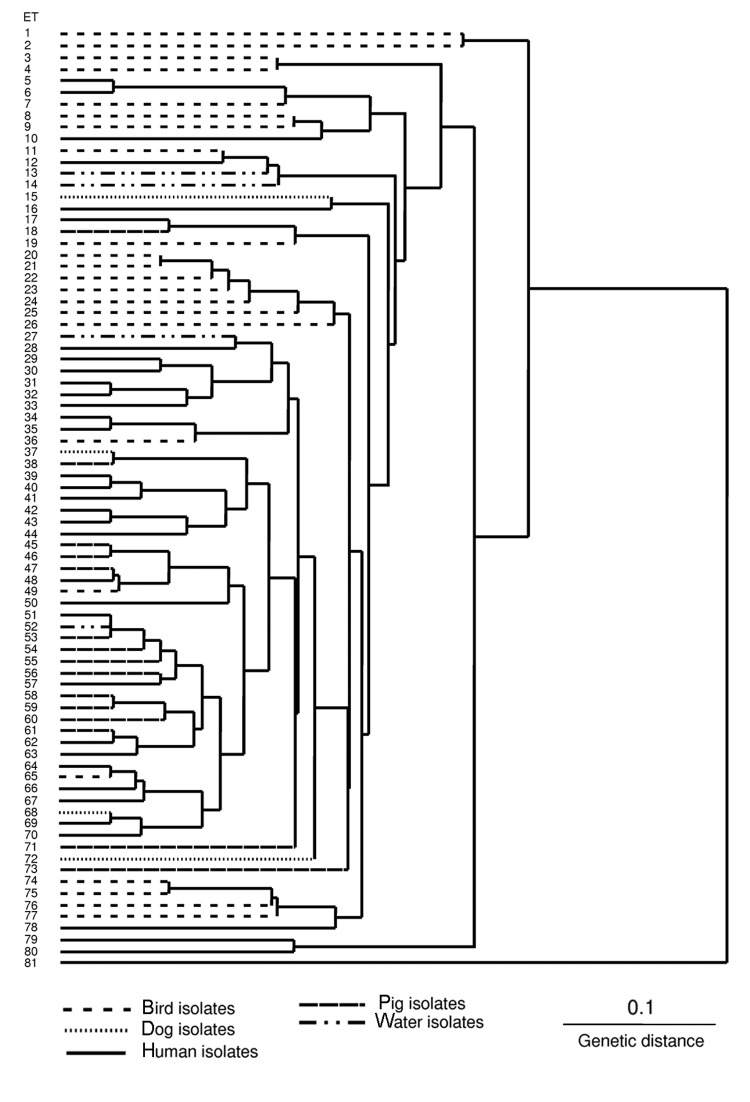
Dendrogram showing relationships between 107 isolates of *Brachyspira pilosicoli* originating from various host species located in electrophoretic types (ETs) 1-80 and *B. aalborgi* NCTC 11492^T^ located in ET81.

Although human isolates did not share an ET with isolates from other species, they were frequently closely related, differing in 1 allele. This occurred with US and Australian pig isolates in ET47 and a human isolate from Oman in ET48; an Australian pig isolate in ET61 and a UK human isolate in ET62; an isolate from an Australian HIV-positive person in ET64, and 1 Dutch and 1 US chicken isolate in ET65; and a Papua New Guinea canine isolate in ET68 and a French human blood isolate in ET69.

The distribution continuum of isolates of diverse host species and geographic origin was consistent with a lack of species specificity and suggests that *B. pilosicoli* isolates naturally have the potential to be transmitted between species. Even should there be some unexpected species-specific barrier preventing "true" animal or bird isolates from colonizing humans, animals have been colonized by human isolates, and thus could act as a reservoir of these for subsequent retransmission to humans. The results suggest that zoonotic transfer of *B. pilosicoli* isolates likely occurs in nature, e.g., after exposure to infected animals or birds, their feces, or contaminated water.
